# The type I insulin-like growth factor regulates the liver stromal response to metastatic colon carcinoma cells

**DOI:** 10.18632/oncotarget.12595

**Published:** 2016-10-12

**Authors:** Maria Celia Fernandez, Roni Rayes, Boram Ham, Ni Wang, France Bourdeau, Simon Milette, Martin lllemann, Nigel Bird, Ali Majeed, Jun Xu, Tatiana Kisselova, Pnina Brodt

**Affiliations:** ^1^ Departments of Surgery, McGill University and the McGill University Health Centre, Montréal, QC, Canada; ^2^ The Finsen Laboratory, Rigshospitalet, Copenhagen, Denmark; ^3^ Biotech Research and Innovation Centre (BRIC), Faculty of Health Sciences, University of Copenhagen, Copenhagen, Denmark; ^4^ Liver Research Group, Clinical Sciences, University of Sheffield, Yorkshire, UK; ^5^ Department of Oncology, School of Medicine, Sheffield Teaching Hospitals, Yorkshire, UK; ^6^ Department of Surgery, University of California, San Diego, La Jolla, CA, USA; ^7^ Medicine, McGill University and the McGill University Health Centre, Montréal, QC, Canada; ^8^ Oncology, McGill University and the McGill University Health Centre, Montréal, QC, Canada

**Keywords:** liver metastasis, tumor microenvironment, tumor stroma, hepatic stellate cells, colorectal carcinoma

## Abstract

Hepatic stellate cells (HSC) play a major role in initiating the liver fibrogenic (wounding) response of the liver and can also orchestrate a pro-metastatic microenvironment in the liver in response to invading cancer cells. Here we explored the role of the hepatic stellate cells in colon carcinoma liver metastasis with emphasis on the contribution of the insulin-like growth factor (IGF) axis to their activation and function. To this end, we used mice with a Tamoxifen inducible liver IGF-I deficiency. We found that in mice with a sustained IGF-I deficiency, recruitment and activation of HSC into tumor-infiltrated areas of the liver were markedly diminished, resulting in decreased collagen deposition and reduced tumor expansion. In addition, IGF-I could rescue HSC from apoptosis induced by pro-inflammatory factors such as TNF-α known to be upregulated in the early stages of liver metastasis. Moreover, in surgical specimens, activated IGF-IR was observed on HSC-like stromal cells surrounding colorectal carcinoma liver metastases. Finally, IGF-targeting *in vivo* using an IGF-Trap caused a significant reduction in HSC activation in response to metastatic colon cancer cells. Therefore, our data identify IGF as a survival factor for HSC and thereby, a promoter of the pro-metastatic microenvironment in the liver. IGF-targeting could therefore provide a strategy for curtailing the pro-metastatic host response of the liver during the early stages of liver metastasis.

## INTRODUCTION

The liver is the most common site of metastases for carcinomas of the gastrointestinal tract. Liver metastases are frequently inoperable and are associated with poor prognosis, resulting in a 25–30% 5-year survival rate for cancers such as colorectal carcinoma [1]. The response of the liver to invading cancer cells is complex and involves various hepatic cells that can together orchestrate a pro-metastatic microenvironment, promoting tumor expansion. A better understanding of the multifaceted response of the liver microenvironment is crucial to designing therapeutic strategies that can curtail or improve cure-rates for hepatic metastases. The hepatic stellate cells (HSC) are key players in the response of the liver to invading cancer cells [2]. In their quiescent state, HSC are located in the space of Disse and identified by the content of vitamin A droplets, the expression of glial fibrillary acidic protein (GFAP) and to a lesser degree, desmin [3–4]. Upon activation, HSC differentiate into myofibroblast-like cells, increasing the expression of desmin and several other markers such as alpha-smooth muscle actin (α-SMA), tissue inhibitors of metalloproteinases (TIMPs) and type I collagen [3, 5–6]. Activated HSC orchestrate the fibrogenic response of the liver to injury, altering the quality and quantity of the extracellular matrix (ECM) and producing the characteristic, type I collagen rich “scar” matrix [7]. The role of HSC in the liver fibrogenic response has been extensively studied, leading to identification of therapeutic targets for the treatment of liver inflammation and fibrosis [8–9]. Their role in liver metastasis, although documented [10], has however, received less attention and is not as well understood.

Tumor cells that metastasize to the liver and invade into the extra-sinusoidal space trigger an inflammatory response that is initiated mainly by Kupffer cells (KCs) and recruited neutrophils that, together, constitute the first line of defense and a major source of cytokine and chemokine production in the tumor microenvironment [11]. Among the factors produced by KC, tumor necrosis factor-α (TNF-α) can either induce apoptosis in sensitive cells or upregulate the expression of pro-metastatic factors such as IL-6 or TIMPs [12] and transforming growth factor-β (TGF-β) that can increase collagen synthesis [13], leading to fibrosis. Activated HSC release various growth factors such as epidermal growth factor (EGF), vascular endothelial growth factor (VEGF), and insulin-like growth factor-I (IGF-I) and metalloproteinases (MMP-2,-9,-13) [3] that together contribute to recruitment of endothelial cells and angiogenesis and promote tumor cell invasion and proliferation [10].

The IGF axis consists of two ligands; IGF-I and IGF-II, their receptors IGF-IR, the IGF-II/mannose 6-phosphate receptor (IGF-II/M6-PR) and IGF-IR-insulin receptor hybrids, and six high affinity IGF binding proteins (IGFBPs). Endocrine IGF-I is produced by the liver under the control of growth hormone and mediates the latter’s growth-promoting functions. Under normal physiological conditions, the IGF-axis plays diverse roles in cellular metabolism, proliferation and differentiation [14]. It has also been implicated in all stages of malignant progression from tumor initiation to metastasis [15–16]. A recent study identified the IGF-I receptor (IGF-IR) as a risk factor for liver metastasis in colorectal carcinoma patients [17], but its precise function in this process remains to be fully understood. *In vitro* studies have shown that IGF-I can promote HSC activation [18] and *in vivo*, the IGF-II/M6-PR was found to be up-regulated on activated HSC during liver fibrosis [19]. However, the role that IGF-I plays in HSC recruitment and activation in response to metastasizing tumor cells remains unknown.

In this study we explored the role of IGF-I in HSC activation during the early stages of colorectal carcinoma liver metastasis, using mice with a Tamoxifen (Tx) inducible liver IGF-I deficiency (iLID). We compared the effect of IGF-I depletion in 2 models; one of an acute IGF-I deficiency that was induced 2 days prior to tumor cell injection (iLID^2D^) and the other of a more sustained IGF-I deficiency induced 3 weeks prior to analysis (iLID^3W^). We found that a sustained (but not a short term) reduction in IGF-I levels markedly decreased the recruitment and activation of HSC in response to metastasizing colon cancer cells with consequences to metastatic expansion.

## RESULTS

### A sustained IGF-I deficiency alters the response of hepatic stellate cells to metastatic colon cancer cells

**Figure 1:**
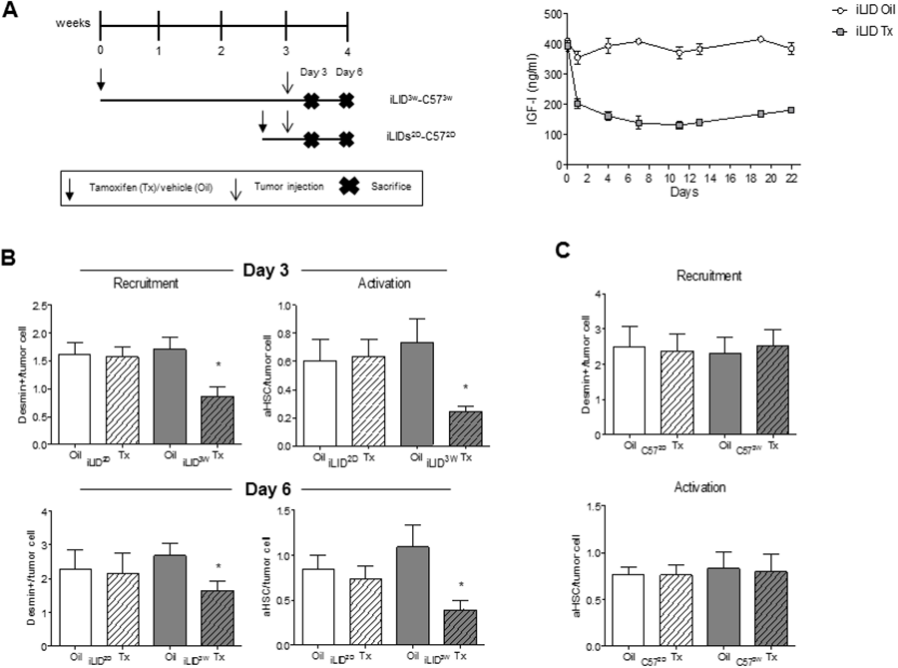
Loss of hepatic stellate cell activation in mice with a sustained liver IGF-I deficiency Shown in **(A-left)** is a schematic representation of the experimental design. MC-38-GFP (5×10^5^) and Tamoxifen (Tx) (0.3 mg/mouse) injections are indicated by arrows and the day the mice were sacrificed by an X. Tx and vehicle (Oil) injected-iLID mice were bled every 2-3 days and IGF-I serum levels measure by ELISA. Results **(A-right)** are expressed as the means (±SEM) of 5-10 serum samples per time point (p<0.05 at all the time points analyzed). Shown in B. and C. are the results of experiments in which iLID (B) or C57Bl/6 (C) mice were injected with Tx or Oil, 2 days (iLID^2D^, C57^2D^) or 3 weeks (iLID^3W^, C57^3W^) prior to tumor injection and the animals sacrificed on day 3 **(B-top)** or 6 (**B-bottom** and **C**) to quantify recruitment and activation of HSC by IHC. For each experiment (B, C), a set of 15 - 20 sections from 3-5 mice per group were analyzed and the number of desmin^+^ or desmin^+^α-SMA^+^(aHSC) cells per tumor cell (x20 objective) were calculated. *p<0.05.

We previously reported that in mice with reduced plasma IGF-I levels from birth [20] or decreased IGF-I bioavailability [21], the incidence of experimental colon carcinoma liver metastases was markedly reduced and this coincided with an altered inflammatory response to the invading tumor cells and increased tumor cell apoptosis, respectively. The objective of the present study was to further investigate the effect of IGF-I depletion on the tumor microenvironment in the liver, in particular its role in regulating the recruitment and activation of hepatic stellate cells, events thought to orchestrate the pro-metastatic host response in the liver. To this end, we used mice with a conditional liver *Igf1* gene deletion induced by a single Tamoxifen (Tx) injection (iLID model). Depletion of liver IGF-I was induced 2 days (short term depletion - iLID^2D^) or 3 weeks (long term depletion - iLID^3W^) prior to tumor inoculation, as depicted in Figure 1A. A reduction in serum IGF-I levels could be observed within 24 hr of Tx injection and was sustained throughout the lives of the mice (Figure 1A). Littermate controls injected with sunflower oil (vehicle) or C57Bl/6 mice injected with Tx were used as controls. Mice were injected with 5×10^5^ colon carcinoma MC-38-GFP cells via the intrasplenic/portal route and sacrificed 3 or 6 days later to quantify and determine the phenotype of HSC recruited into the tumor-infiltrated areas. In control mice, we observed an accumulation of activated HSC in areas invaded by the tumor cells as early as 3 days post tumor cell inoculation and this was also seen in iLID^2D^ mice (Supplementary Figure S1). In iLID^3W^ mice, however, the recruitment and activation of tumor-associated HSC were significantly reduced, as was also evident 6 days post tumor injection (Supplementary Figure S1, Figure 1B). To rule out any non-specific effects of Tx, HSC were also analyzed in tumor-injected C57Bl/6 mice that were pre-treated with Tx, 2 days or 3 weeks earlier. In these mice, we observed no change in the recruitment or activation status of HSC relative to controls as measured 6 days post tumor injection (Figure 1C).

### Reduced tumor colony size in mice with a sustained IGF-I deficiency

**Figure 2:**
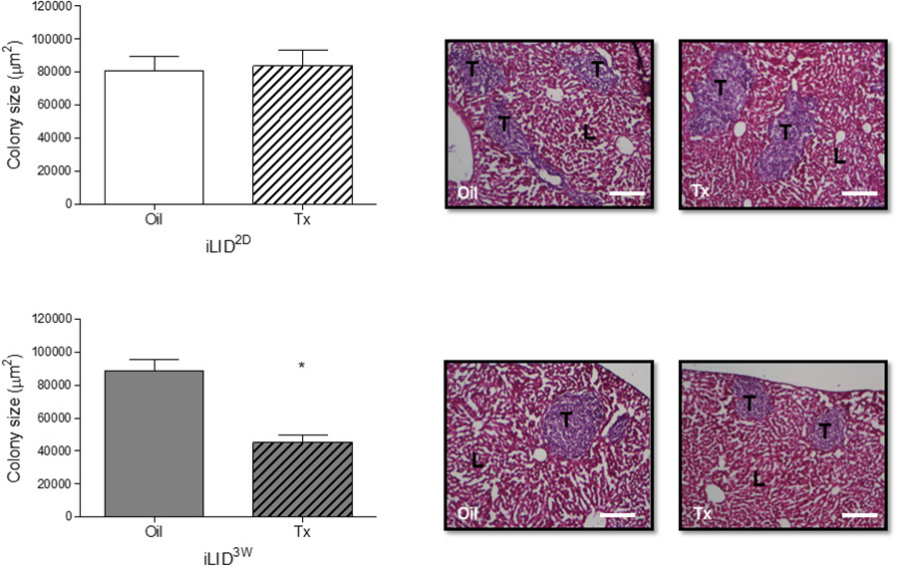
Decreased growth of liver metastases in mice with a sustained IGF-I deficiency iLID mice (n=3-5) were injected with Tx or Oil 2 days (iLID^2D^) or 3 weeks (iLID^3W^) prior to the intrasplenic/portal injection of 5×10^5^ MC-38 cells, sacrificed 6 days later and FFPE liver sections prepared and H&E stained to quantify tumor colonies. Images on the right are representative of a set of 15 - 20 sections per group. Total surface area occupied by tumor cells (bar graph, left) was quantified by Image J (x10 objective, scale bar = 200μm). T: Tumor, L: Liver; *p<0.05.

To determine the effect of reduced HSC recruitment on the growth of the hepatic metastases, mice were injected with 1×10^5^ MC-38 cells via the intrasplenic/portal route, livers removed 6 days later and formalin fixed, paraffin embedded (FFPE) sections prepared and H&E stained. We observed a significant reduction in colony size in iLID^3W^ mice, as compared to oil-injected controls, but this was not observed in iLID^2D^ mice (Figure 2). These results suggested the following: 1. that the reduced levels of circulating IGF-I (of equal magnitude in iLID^2D^ and iLID^3W^ mice) was not itself sufficient to directly inhibit tumor cell growth in the liver, and 2. that the impaired recruitment and activation of HSC in the tumor microenvironment resulted in a reduction in the growth of metastases.

### HSC-derived collagen production is decreased in mice with a sustained reduction in circulating IGF-I levels

**Figure 3:**
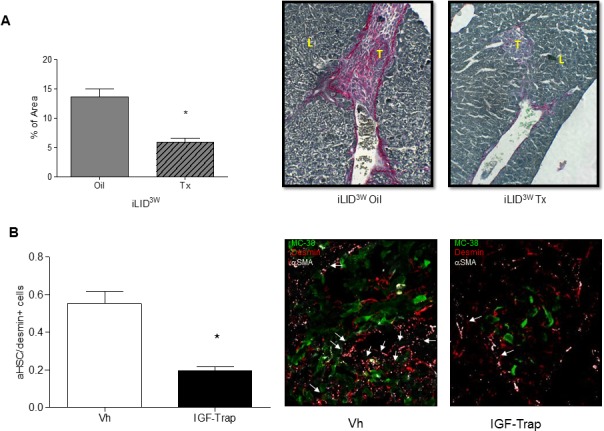
A sustained IGF-I deficiency reduces collagen production in tumor-injected mice and treatment with an IGF-I inhibitor impairs HSC activation iLID mice were injected with Tx or Oil 3 weeks (iLID^3W^) prior to intrasplenic/portal injection of 5×10^5^ MC-38 cells. FFPE sections of the livers obtained 6 days post tumor injection were stained with Sirius Red and the red-stained areas quantified in a total of 20-50 fields derived from 3 - 4 animals per condition (x10 objective). Shown in **(A-right)** are representative images of Sirius Red –stained sections and in **(A-left)** results of the quantification performed by Image J expressed as the % of total surface area/field that was stained red (collagen). In a separate experiment (B). C57Bl/6 mice injected with 5×10^5^ MC-38-GFP were treated i.v. with 10 mg/kg IGF-Trap or saline (Vh) as control, on days 2 and 4 post tumor injection and mice sacrificed on day 6. Activated HSC (desmin^+^α-SMA^+^) recruited into tumor-infiltrated areas were quantified and the results are expressed as % of all desmin^+^ cells/field in a set of 15-20 sections obtained from 3-5 animals per group (x40 objective) **(B-left)**. Representative IHC images of tumor-infiltrated areas are shown on the right. Arrows denote activated hepatic stellate cells (aHSC); L- Liver, T- Tumor; *p<0.05

HSC contribute to metastatic expansion by depositing ECM collagen and thereby promoting endothelial cell migration, angiogenesis and tumor cell invasion [22]. In livers of iLID^3W^ mice injected with colon carcinoma MC-38 cells, we observed a reduction in collagen production as quantified by Sirius Red staining [23] (Figure 3A). To confirm that the observed reductions in HSC activation and ECM deposition in iLID^3W^ mice were a direct consequence of IGF-I depletion and not the result of reduced tumor growth, we also analyzed HSC activation in a tumor - free model namely, the CCl_4_-induced liver fibrosis model known to be driven by activated HSC [24]. When collagen deposition in this model was quantified by Sirius Red staining, a similar reduction in HSC-mediated collagen deposition was observed in iLID^3W^ mice, but this was not seen in iLID^2D^ mice, as compared to their respective controls. This was also confirmed by qPCR for α-SMA and collagen 1α1 (Supplementary Figure S2A and S2B). Moreover, treatment of wild-type mice with an IGF-IR targeting inhibitor, the IGF-Trap, that reduces IGF-I bioavailability and IGF-IR signaling *in vivo* [21], also significantly reduced HSC activation in response to tumor cells (Figure 3B).

### GH can compensate for reduced IGF-I production in mice with a short term IGF-I deficiency

**Figure 4:**
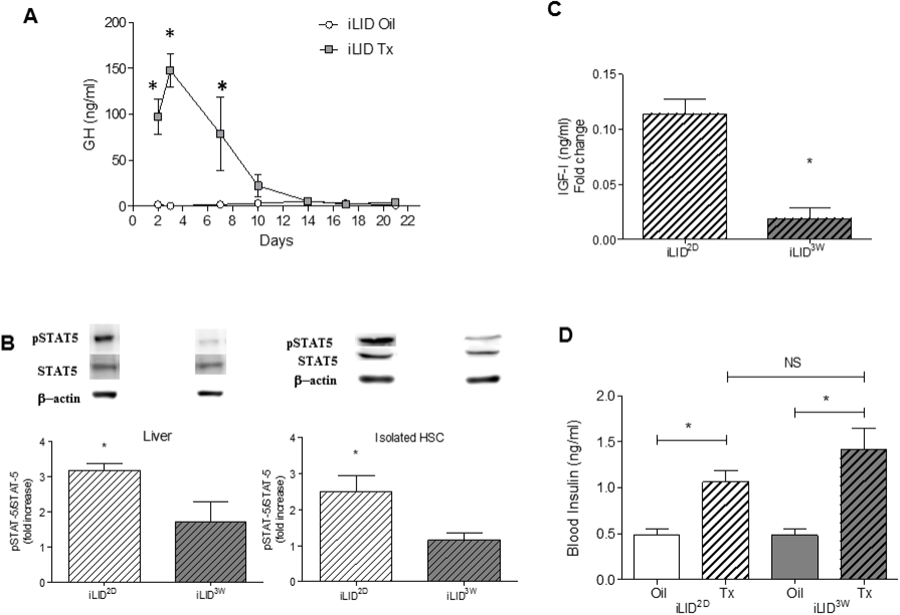
Increased GH production and signaling in mice with short term IGF-I depletion iLID mice were injected with Tx or Oil and blood was collected twice weekly for 3 weeks. GH **(A)** was measured by ELISA and the results are expressed as the means (±SEM) of 5-10 serum samples per time point. Proteins were extracted from livers or from isolated HSC obtained from 4 - 6 iLID^2D^ or iLID^3W^ mice (and respective controls) and analyzed by Western blotting **(B)** using the indicated antibodies (Supplementary Table S1). Representative blots for pSTAT-5, STAT5 and β-Actin that was used as a loading control are shown on top of the bar graphs. Results are expressed as mean (±SEM) fold increase relative to the respective control (n=6). IGF-I levels in cell lysates of isolated HSC **(C)** were measured using an ELISA. The results are expressed as mean (±SEM) concentration detected in the indicated cells (n=3). Blood insulin levels **(D)** were measured in the indicated mice after a 6 hr fasting using an ELISA. The results, expressed as ng/ml are means (±SEM) of 5-10 serum samples per time point. NS, not significant *p<0.05

Our results suggested that there was a fundamental difference between the hepatic microenvironments in mice with a short versus sustained IGF-I depletion. To gain insight into the underlying cause(s), we first measured serum levels of insulin and GH, because their production is regulated by serum IGF-I levels [25]. In the iLID mice, we found a significant increase in circulating GH levels from day 2 and up to one week post Tx injection, at which time the levels normalized to those observed in controls (Figure 4A). This was not due to a recovery in circulating IGF-I levels which remained low for the entire experimental period, as shown in Figure 1A. During the first week post Tx injection, the increase in circulating GH levels was also reflected in a significant increase in the levels of phosphorylated STAT5 (pSTAT5), a transcription factor activated downstream of GH signaling, as seen in both total liver extracts and in HSC isolated from iLID mice, 2 days post Tx injection (Figure 4B). STAT5 regulates IGF-I expression levels [26]. Indeed, we observed that in HSC obtained from iLID^2D^ mice, IGF-I production levels were significantly higher than in HSC from iLID^3W^ mice (Figure 4C), suggesting that autocrine IGF- signaling may be triggered in the former cells. To rule out a direct effect of GH on the tumor cells in iLID^2D^ mice that could result in the production of pro-fibrogenic factors that activate HSC in a paracrine manner, MC-38 cells were treated with rhGH *in vitro* and qPCR used to analyze changes in growth factor expression. We found that expression levels of the pro-fibrogenic factors TGF-β, CTGF and PDGF were unaffected by GH stimulation (Supplementary Figure S3), suggesting that tumor-derived factors were not contributing to HSC activation in the presence of high circulating GH levels. As expected, IGF-I expression in the tumor cells did increase in response to GH, potentially contributing to the stimulation of HSC in iLID^2D^ mice.

Taken together, these results suggested that the difference in the HSC response in these mice was due, at least in part, to a potential compensatory effect of GH in mice with a short term, but not a sustained, liver IGF-I depletion. Of interest, we observed an increase in serum insulin levels in iLID mice when analyzed at 2 days or 3 weeks post Tx injection (Figure 4D), suggesting that the divergent HSC activation levels and differences in collagen production and tumor growth observed in these mice were not likely due to differences in insulin production levels and metabolism.

### A sustained reduction in circulating IGF-I levels affects IGF-IR expression and activation levels in HSC

**Figure 5:**
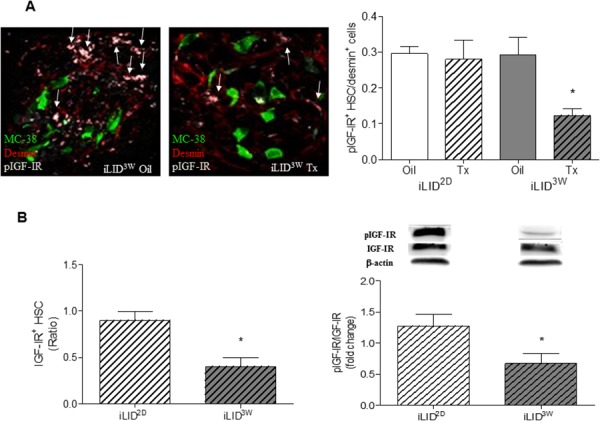
Decreased IGF-IR levels and reduced IGF-IR activation in hepatic stellate cells obtained from mice with a sustained IGF-I deficiency In FFPE sections derived from tumor cell injected mice, phospho-IGF-IR (pIGFIR) levels were analyzed by IHC **(A)** and the proportion of pIGF-IR^+^desmin^+^ cells in the total desmin^+^ population/field were calculated. Results in the bar graph (right) are based on a set of 15 - 20 sections derived from 3-5 mice per group (x40 objective) and are expressed as means (±SEM). Representative IHC images are shown on the left. Arrows denote pIGFIR^+^ HSC. HSC were isolated from iLID^2D^ or iLID^3W^ mice (and respective controls) and analyzed by flow cytometry **(B-left)** or Western blotting **(B-right)** using antibodies to the α subunit of IGF-IR and pIGF-IR, respectively and β-actin as a loading control. Results of flow cytometry are means (±SEM) of 3 analyses and are expressed as a ratio to the respective vehicle-injected controls that were assigned a value of 1. Results of the WB analysis are based on 6 samples and are expressed as mean fold change (±SEM) relative to the respective controls that were assigned a value of 1. Results of a representative immunoblot are shown on the top. *p<0.05.

The above results implicated IGF-I in HSC activation in response to invading tumor cells. In HSC surrounding metastatic tumor cells in the liver, we found high levels of activated IGF-IR and this was markedly reduced in iLID^3W^, but not iLID^2D^ mice (Figure 5A). Moreover, when HSC were isolated from iLID mice and IGF-IR expression and activation levels analyzed by flow cytometry and Western blotting, we found a significant reduction in IGF-IR expression and activation in HSC isolated from iLID^3W^, but not iLID^2D^ mice as compared to the respective controls (Figure 5B). The data suggest that in mice with a short term IGF-I deficiency, HSC may compensate for lower circulating IGF-I levels by increased GH-induced autocrine IGF signaling. This compensatory mechanism is not, however, available to HSC exposed to a sustained reduction in hepatic IGF-I levels as GH levels decline overtime (See proposed model in Figure 8).

### IGF-I is a survival factor for HSC subjected to the pro-apoptotic effects of inflammatory cytokines

**Figure 6:**
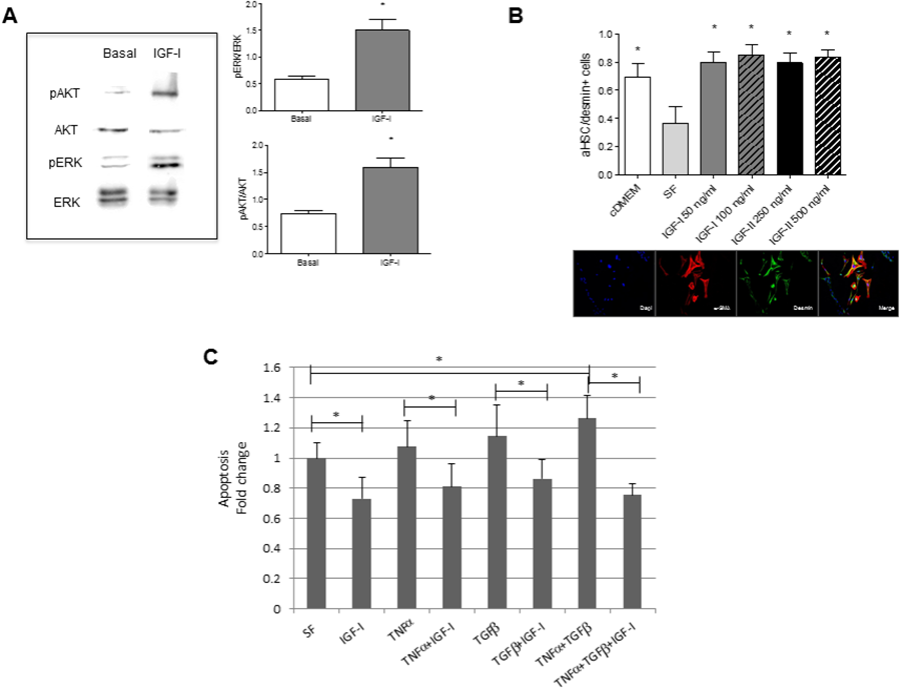
Increased IGF-IR signaling, activation and survival in HSC stimulated by IGF-I *in vitro* HSC were isolated from normal C57Bl/6 female mice (n=6) and cultured in collagen coated (5μg/cm^2^) 6 - **(A)**, 8- **(B)**, or 24- **(C)**, well plates. Western blotting with the indicated antibodies (A) was performed on total cell lysates following stimulation of the HSC for 5 minutes with 100 ng/ml rmIGF-I and the bands analyzed by laser densitometry and quantified using the Image J software. Shown on the left are the results of a representative immunoblot out of 3 performed and in the bar graphs the means (±SEM) of 3 separate experiments. To analyze HSC activation (B) or apoptosis (C), the cells were cultured first for 24 h in medium containing 10% FBS DMEM (cDMEM), and then treated (or not) for 3 days (B) or 24 hr (C) with rmIGF-I, rmIGF-II, TNF-α and TGF-β at the indicated concentrations in serum free medium (SF). Cells (B) were fixed with a methanol: acetone (1:1) solution and stained with DAPI (blue), and antibodies to α-SMA (red) and desmin (green). Representative images are shown on the bottom. Apoptosis (C) was measured using the Cell Death ELISA kit. Results in the bar graph are based on measurements of absorbance (at 405 nm) in triplicate samples derived from two independent experiments and are expressed as means (±SEM) of fold change relative to serum starved cells that were assigned a value of 1. *p<0.05

To test the effect of IGF-I on HSC more directly, we isolated quiescent, vitamin A^+^ HSC from the livers of wild type mice. We confirmed that treatment of the cultured cells with 100 ng/ml IGF-I for 5 min activated both the PI3K/AKT and MEK/ERK signaling pathways (Figure 6A) known to be engaged downstream of the IGF-IR [27]. HSC cultured in the presence of IGF-I or IGF-II had significantly increased α-SMA expression levels, as assessed by immunohistochemistry (Figure 6B), suggesting that these ligands could directly activate the cells. Quiescent HSC are located in proximity to hepatocytes – the major source of endocrine IGF-I. This suggests that under conditions of homeostasis, they are insensitive to IGF-I induced activation but that they may become sensitized under pathological conditions such as inflammation and fibrogenesis. Both acute and chronic liver diseases are characterized by elevated serum levels of TNF-α and TGF-β. We asked whether IGF-I can attenuate potential pro-apoptotic effects of these factors and in this way, provide a survival advantage to HSC under these pathological conditions. We found that freshly isolated murine HSC underwent apoptosis in the presence of TNF-α and TGF-β, when administered alone or in combination. These cells were, however, rescued from cell death in the presence of IGF-I (Figure 6C), suggesting that under conditions of liver injury, IGF-IR may be engaged and act as a survival factor for HSC (see Figure 8).

### IGF-IR is phosphorylated in activated stromal cells surrounding hepatic metastases in colorectal carcinoma patients

**Figure 7:**
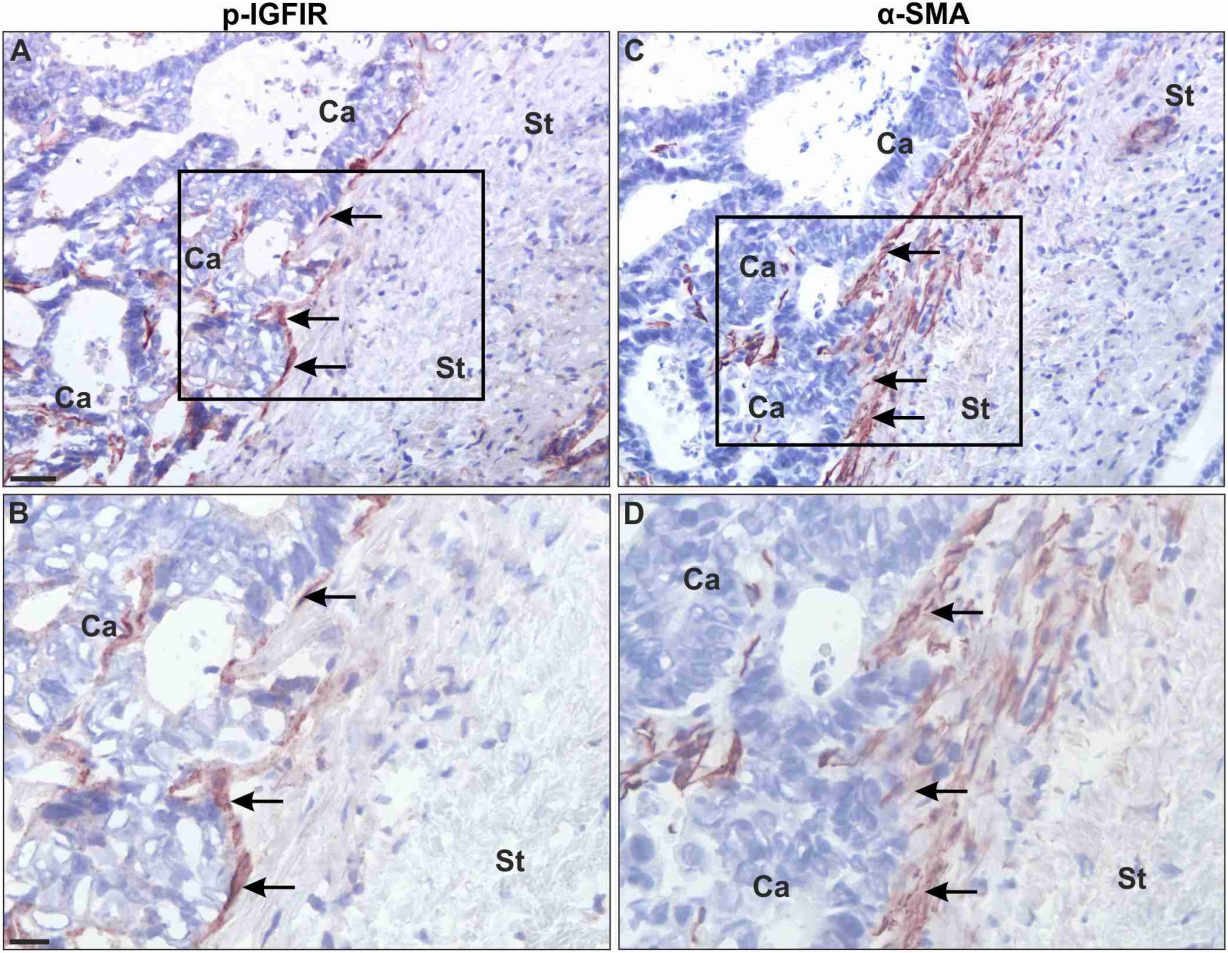
Myofibroblasts at the periphery of colorectal carcinoma liver metastases express activated IGF-IR Adjacent frozen tissue sections were processed for single immunoperoxidase staining using a polyclonal Ab to pIGFIR **(A, B)** or an anti- α-SMA Mab **C, D**. The antibodies were visualised with NovaRed. Black squares in (A) and (C) represent the area shown in higher magnification in (B, D). Shown (C, D) are representative images from one of 6 livers analyzed. In all the specimens examined, the majority (over 80%) of the immunoreactivity for pIGFIR was seen in spindle-shaped cells (arrows in A, B) at the rim of the metastases (indicated with Ca) in the tumor-stromal interface (indicated by St). These spindle-shaped cells were identified by staining adjacent sections for α-SMA (a marker of myofibroblasts). All pIGFIR-positive spindle-shaped cells were α-SMA^+^, identifying them as myofibroblasts (arrow in C, D). pIGFIR immunoreactivity was also observed in polymorphonuclear cells (presumably neutrophils) close to the metastatic lesions (data not shown). Bars: ∼40 μm (A, C) and ∼20 μm (B, D).

Finally, to determine the broader relevance of our findings, we analyzed by IHC surgical resections from chemo-naive colorectal carcinoma patients that underwent partial hepatectomy to remove liver metastases. Cryostat sections were immunostained with antibodies to α-SMA and pIGF-IR to identify activated HSC in which IGF-IR signaling was induced. In the stromal interface at the edge of the metastases, multiple spindle-shaped cells co-expressing α-SMA and pIGFIR could be identified (Figure 7), implicating the IGF-IR in the stromal response of the liver to CRC metastases in the clinical disease.

## DISCUSSION

Our results show that in a liver microenvironment altered by a sustained IGF-I deficiency, HSC recruitment and activation during the early stages of colon cancer metastasis are diminished, resulting in reduced growth of metastatic tumor cells. Taken together, they implicate the IGF-IR/IGF axis in the regulation of HSC survival and activation and thereby, in orchestrating the pro-metastatic response of the microenvironment to invading cancer cells. HSC activation and the growth of metastases are inter-dependent. Our finding of reduced HSC activation and type I collagen production in a tumor-free model of liver injury induced in iLID^3W^ mice by CCl_4_ suggests that the reduction in HSC activation in mice with a sustained IGF-I depletion was the consequence rather than the cause of reduced metastasis, indicating that the IGF axis is involved in the wounding response of the liver, independently of the trigger. In the context of metastasis, a reduction in type I collagen production could result in reduced endothelial cell migration, loss of angiogenesis and reduced tumor invasion [22], all leading to reduced tumor expansion, as seen in our model. Our results suggest that the reduction in HSC activation was associated with reduced IGF-IR expression levels on HSC, implying that physiological IGF-I levels are required to maintain IGF-IR expression levels on these cells. Interestingly, a similar reduction in IGF-IR expression levels in neutrophils was reported in mice with a sustained (but not short term) blockade of IGF-IR signaling, mediated by the administration of an anti IGF-IR antibody (AMG-479) for 3 weeks [28].

**Figure 8:**
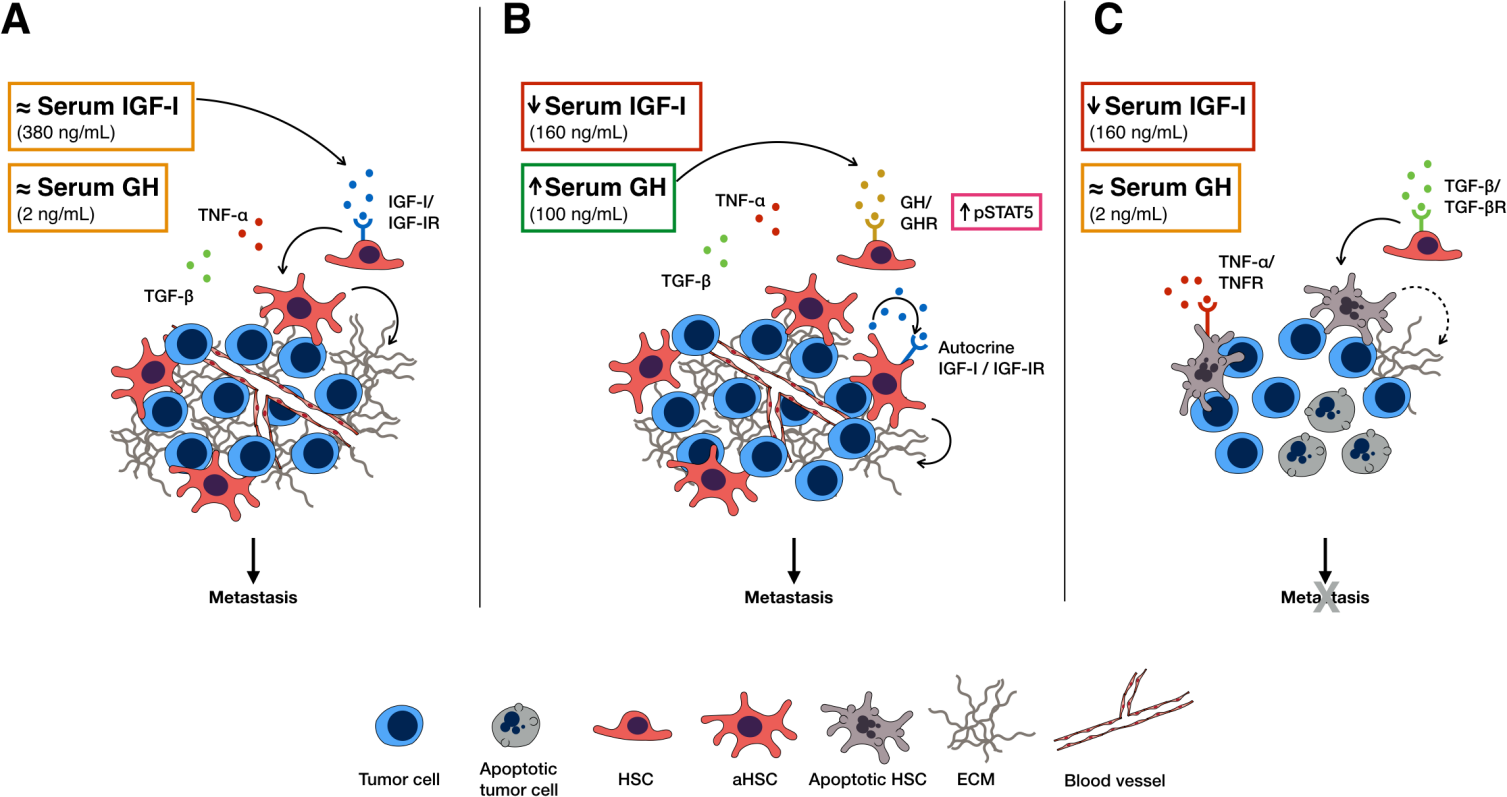
A model, postulated based on our data, for the role of IGF-I in HSC survival and activation and the effects of IGF-I depletion on CRC liver metastasis In the presence of physiological serum IGF-I levels **(A)** HSC can be rescued from inflammation- induced apoptosis and are activated in response to tumor cell invasion, altering the microenvironment by ECM deposition that promotes angiogenesis and tumor expansion. Under conditions of short-term serum IGF-I depletion **(B)** serum GH levels are transiently increased and may compensate for the lack of IGF-I by triggering autocrine IGF-I/IGF-IR signaling in HSC via STAT5 signaling, thereby rescuing them from apoptosis. Under conditions of sustained IGF-I depletion **(C)** as serum GH levels normalize, the HSC become more susceptible to TGF-β and TNF-α- induced apoptosis and can no longer contribute to the pro-metastatic microenvironment required for tumor growth.

In mice with a short term IGF-I deficiency, no reductions in HSC recruitment or the growth of metastases were observed. Our results suggest that in these mice, GH levels were transiently increased, likely as a feedback response to reduced IGF-I bioavailability [25]. Moreover, IGF-I production in the HSC was increased and this could have resulted in autocrine IGF-IR activation that could, in turn, compensate for loss of liver IGF-I production and maintain IGF-IR expression levels (A postulated model is depicted in Figure 8). This mechanism appears to be lacking in HSC exposed to a sustained reduction in IGF-I levels, partially because GH levels return to their basal levels within a week post Tamoxifen treatment and also because of their reduced IGF-IR expression levels. The differences in the outcome of IGF-I deficiency in these two models also indicate that the effect of reduced circulating IGF-I on metastatic expansion was, at least in part, indirect acting via changes to the tumor microenvironment.

IGF-I is a mitogenic and survival factor for various cell types and these effects are mediated via the MEK/ERK and PI3K/AKT pathways, respectively [27]. Indeed, we show here that these pathways were activated in isolated HSC following a brief exposure to IGF-I. The increased activation of HSC cultured in the presence of IGF-I (or IGF-II) as reflected in upregulated α-SMA expression is consistent with findings by others that implicated the ERK and AKT pathways in increased type I collagen synthesis in IGF-I-stimulated human HSC [29]. Experimental models of fibrosis have provided conflicting results on the role of IGF-I in this process. For example, IGF-I therapy was shown to improve liver fibrosis in a bile duct ligation (BDL) model, where the underlying cause is cholestasis [30]. Recent studies have, however, shown that portal fibroblasts and not HSC are the major players in this model of liver injury [24], suggesting that this process may be driven by other factors.

In normal livers, HSC can be exposed to hepatocyte-derived IGF-I, yet remain quiescent. This suggests that the role of IGF-I is complex and that other factors may be involved in generating conditions permissive for IGF-induced HSC activation. The entry of metastatic tumor cells into the liver has been shown to trigger a rapid release of pro-inflammatory cytokines including TNF-α [31]. Tumor cell invasion also appears to recapitulate some aspects of the liver wounding response triggering HSC activation, ECM deposition and the release of growth factors such as TGF-β, EGF, VEGF and IGF-I. Our data suggest that HSC may be susceptible to apoptosis in this microenvironment rich in TNF-α and TGF-β and that IGF-I may act as a survival factor rescuing these cells from death and promoting their recruitment and activation.

In surgical specimens of colorectal carcinoma liver metastases, we observed stromal cells co-expressing α-SMA and pIGFIR in the periphery of the metastatic tumors, consistent with our findings in the mouse model. Other types of stromal cells such as bone marrow derived fibrocytes and portal fibroblasts have been shown to play a role in the fibrogenic response of the liver [32] and may also be involved in the hepatic response to liver metastases [33]. While our data cannot definitively identify these cells as HSC in origin, they do strongly implicate IGF-IR in the response of the liver microenvironment to metastatic CRC.

To our knowledge, our study is the first to show that IGF-I plays a direct and essential role in HSC survival and activation in the context of liver metastasis, thereby identifying this axis as a target in the tumor microenvironment. Taken together with our previous findings that an IGF-Trap could markedly reduce colon cancer liver metastasis [21], the present data suggest that this effect was mediated, at least in part, through modulation of the pro-metastatic liver response to disseminating cancer cells.

## MATERIALS AND METHODS

### Ethics statement

Investigation has been conducted in accordance with the ethical standards and according to the Declaration of Helsinki and according to national and international guidelines and has been approved by the authors' institutional review board.

### Animals

All mouse experiments were carried out in strict accordance with the recommendations of the Canadian Council on Animal Care (CCAC) “Guide to the Care and Use of Experimental Animals” and under the conditions and procedures approved by the Animal Care Committee of McGill University (AUP # 5260). C57Bl/6 female mice were obtained from Charles River Laboratories (St. Constant, QC, Canada) and used for the experiments at the age of 7-10 wks. Mice with an inducible, liver specific IGF-I deficiency (iLID) were generated and their phenotype confirmed as described in detail previously [20]. Briefly, in these mice, a single Tamoxifen (Tx) injection results in gene recombination by activating the Cre recombinase gene under the control of a liver specific anti-trypsin 1 α promoter. The inducible *igf1* deletion results in a ∼60% reduction in serum IGF-I levels within ∼18 hr of a single i.p. injection of 0.3 mg Tx per mouse [34]. iLID breeder mice were originally obtained as a gift from Dr. Shoshana Yakar (New York University, NY) and a colony was maintained at the McGill University Health Center Animal facility, as per the guidelines of the McGill University Animal Care Committee. Female iLID and C57Bl/6 mice were injected either with Tx or with the vehicle, sunflower oil, as control [20, 34].

### Cells

The murine colon carcinoma MC-38 cells were originally from an NCI repository and were obtained as a kind gift from Dr. Shoshana Yakar (New York University, NY) in 2005. They were recently authenticated by Didion and colleagues using SNP profiling as described [35]. The cells were transfected with a pEGL plasmid expressing the Green Fluorescent Protein (GFP) and maintained in DMEM supplemented with 10% FBS, as described elsewhere [36]. The cells were periodically tested for mouse and human pathogens and mycoplasma infection, as per the McGill University Animal Care committee and the McGill University Biohazard committee guidelines, and were last confirmed to be pathogen and mycoplasma free in 2015. To avoid cross-contamination and phenotype changes, these cells were maintained as a frozen stock and cultured for only 2 - 4 weeks prior to use in the experiments. Authentication of these cell lines based on morphology and metastatic phenotype was performed regularly, and no phenotype changes were observed throughout the duration of this study.

### Antibodies and reagents

All antibodies used in this study are listed in Supplementary Table S1. Carbon tetrachloride (CCl_4_) and sunflower oil were purchased from Sigma (St. Louis, MO).

### Experimental metastasis assays

Experimental hepatic metastases were generated by the intrasplenic/portal injections of 5×10^5^ MC-38-GFP cells in 100 μl of DMEM followed by splenectomy 1 min later, as we described in detail previously [37–38].

### IHC and confocal microscopy

Following the specified treatment, the livers were perfused, first with PBS and then with 4 ml of a 4% paraformaldehyde solution. The perfused livers were placed in 4% paraformaldehyde for 48 hr and then in 30% sucrose for an additional 48 hr, before they were stored at −80°C. For IHC, 10 μm cryostat sections were prepared, treated with a blocking solution (1% bovine serum albumin (BSA), 1% fetal bovine serum (FBS) in PBS) and then incubated overnight at 4°C with the primary antibodies used at the indicated dilutions (see Supplementary Table S1) and followed by the appropriate Alexa Fluor conjugated secondary antibodies for 1 hr at RT. Sections stained with the secondary antibodies only were used as controls in all the experiments. Tumor infiltrated areas were identified based on GFP expression and HSC identified based on the expression of the cell surface markers desmin and α-SMA. For immunodetection of desmin, two different antibodies were used (see Supplementary Table S1) to ensure antigen specificity. The sections were mounted in the Prolong Gold anti-fade reagent (Molecular Probes, Eugene, Oregon, USA) and confocal images were captured with a Zeiss LSM 780 microscope with a spectrum detection capability. Immunostained cells were quantified blindly using the Image J software in 30-50 images acquired per section.

### Liver fibrosis model

Liver fibrosis was induced by intra-gastric gavage with 200 μl of 25% carbon tetrachloride (CCl_4_) in sunflower oil, twice weekly for 6 weeks [23]. Formalin-fixed livers were stained with Sirius Red and the fibrotic surface area quantified using the Image J software. Liver fragments were snap frozen and stored at −80°C for RNA extraction and qPCR analysis as described below.

### Quantitative real time RT-PCR

RNA was extracted from liver tissue or MC-38 cells using the Trizol reagent (Life Technologies, Inc., Burlington, Ontario, Canada) according to the manufacturer’s instructions. One μg of total RNA was reverse transcribed as described previously [31] and quantitative real-time RT-PCR was performed in a BioRad LightCycler (BioRad, Hercules, CA, USA) using SYBR (Roche, ON, Canada) and the primers listed in Supplementary Table S2. The Iq5 software was used for data analysis.

### IGF-Trap treatment

C57Bl/6 female mice were injected i.v. with 10 mg/kg of the IGF-Trap [21] (or vehicle for control), 2 and 4 days following the intrasplenic/portal inoculation of 5×10^5^ MC-38 cells. Livers were processed for IHC, 6 days post tumor injection.

### Measurement of blood GH, IGF-I and insulin

Blood was collected from mice 6 hr after fasting. Serum insulin and GH levels were measured using the Mercodia mouse insulin ELISA Kit (Mercodia AB, Uppsala, Sweden) and the rat/mouse GH ELISA Kit (EMD Millipore, ON, Canada), respectively. IGF-I levels in the serum or in protein extracts were analyzed using the Mouse/Rat IGF-I Quantikine ELISA Kit (R&D Systems, Minneapolis, MN, USA).

### Isolation of HSC

HSC were isolated by Percoll density gradient centrifugation following liver perfusion and consecutive digestion with 0.2% pronase and 0.05% collagenase/pronase solutions, as described previously [23, 39]. Isolated HSC were cultured in collagen coated plates (5μg/cm^2^, Roche, Basel, Switzerland) with 10% FBS enriched DMEM for the indicated duration or used for protein extraction, as described below. Isolated HSC were stimulated with 50 or 100 ng/ml recombinant mouse IGF-I (R&D Systems, Minneapolis, MN, USA) or with 250 or 500 ng/ml rhIGF-II (BioVision, San Francisco, CA, USA) and the medium replenished daily for 3 days, before the cells were fixed in a chilled acetone:methanol solution (1:1) and immunostained with anti- desmin and/or α-SMA antibodies. Apoptosis induced by serum starvation, TNF-α (10 ng/ml) or TGF-β (100 pM) was measured using the Cell Death detection ELISA (Roche, ON, Canada), as instructed by the manufacturer. Prior to analyzing activation of IGF-I signaling, HSC were stimulated for 5 minutes with 100 ng/ml rmIGF-I (R&D Systems, Minneapolis, MN, USA).

### Western blot analysis

Livers or cells were lysed with RIPA buffer (25 mM Tris-HCl pH 7.6, 150 mM NaCl, 1% Triton X-100, 1% sodium deoxycholate, 0.1% SDS) containing a protease inhibitor cocktail (Roche, Basel, Switzerland). The lysate proteins were separated by SDS-PAGE and Western blotting performed as previously described [40]. Primary and secondary antibodies were used at the dilutions detailed in Supplementary Table S1. Densitometry was performed using the Image Quant LAS 4000 image analysis system (GE Healthcare, ON, Canada) and the images analyzed using the Image J software.

### Flow cytometry

IGF-IR expression levels in isolated, vitamin A – containing HSC were analyzed by flow cytometry using the FACSCanto II RUO system (BD Biosciences, ON, Canada). Gating was based on vitamin A positivity, as detected by violet laser (405 nm), and IGF-IR positivity [24].

### Patient material

All samples were obtained from patients undergoing partial hepatectomy for colorectal cancer liver metastases at the Northern General Hospital, Sheffield, UK, following signed informed consent. Specimens were obtained in the operating theatre, sampled at the tumor/liver interface, frozen in isopentane/dry-ice within 30 min post resection, embedded in O.C.T. (Sakura Finetek, Tokyo, Japan) and stored in liquid nitrogen until analyzed. The group comprised of 4 males and 2 females, all of whom developed metachronous metastases. The protocol was approved by the Bradford Research Ethics Committee (08/H1302/73, Sheffield Teaching Hospitals Research Office ref: 14958).

### Immunohistochemistry

For analysis of patients’ specimens five μm thick cryostat sections were fixed in neutral buffered 4% formalin at RT for either 5 (p-IGFIR) or 30 (α-SMA) min. Endogenous peroxidase activity was blocked by three 15 min incubations in 1% H_2_O_2_. The sections were washed in Tris-buffered saline (TBS, 50mM Tris-HCL, 150 mM NaCl) containing 0.5% Triton X-100 (TBS-T) and placed in Shandon racks with immunostaining cover plates (Thermo Shandon, Pittsburgh, PA) for further incubation. Antibodies were diluted in Antibody Diluent with Background-Reducing Components (S3022, Dako) to concentrations of 10 (p-IGFIR, Abcam) and 0.35 (α-SMA, Dako) μg/ml and incubated overnight at 4°C. Primary antibodies were detected with EnVision^™^ Horseradish Peroxidase rabbit or mouse reagents. Each incubation step was followed by washes in TBS-T. The sections were developed with NovaRed (Vector laboratories, Burlingame, CA) and counterstained in Mayer’s haematoxylin. Finally, slides were dehydrated and mounted using a Dako Coverslipper.

### Statistical analysis

The one- or two-way ANOVA was used to analyze differences in HSC recruitment or activation and for all the *in vitro* data. The student t-test was used to analyze all other *in vivo* data, as well as Western blot and ELISA results. Statistical significance was defined as a p-value of less than 0.05.

## SUPPLEMENTARY MATERIALS FIGURES AND TABLES


