# Supplementary material for: The type I insulin-like growth factor regulates the liver stromal response to metastatic colon carcinoma cells
Source: Oncotarget. 2016 Oct 12;8(32):52281–93. doi: 10.18632/oncotarget.12595 (PMC5581028; doi:10.18632/oncotarget.12595)
Supplement: Supplementary file 1 [file oncotarget-08-52281-s001.pdf]

The type I insulin-like growth factor regulates the liver stromal response to metastatic colon carcinoma cells

SUPPLEMENTARY FIGURES AND TABLES

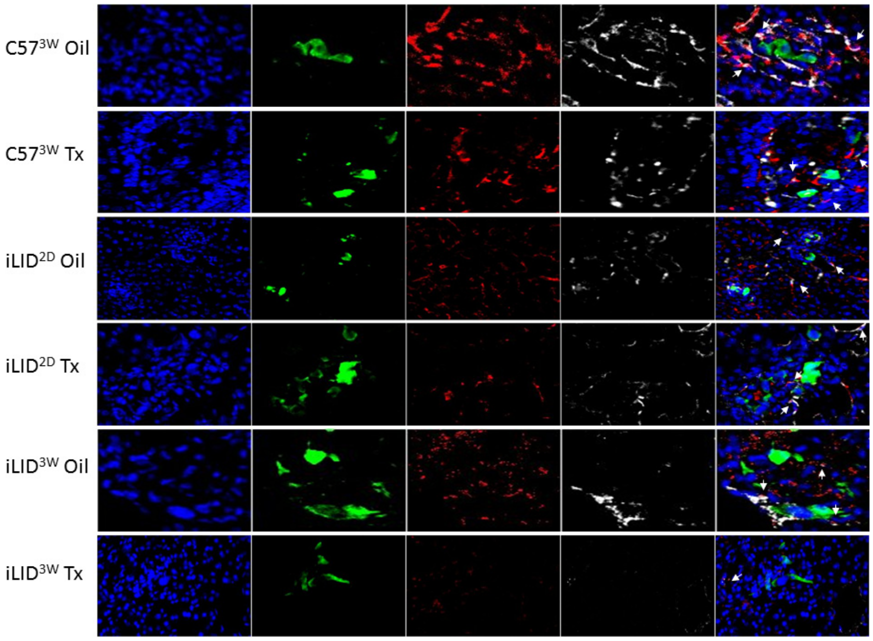

Supplementary Figure S1: Reduced hepatic stellate cell recruitment and activation in mice with a sustained liver IGF-I deficiency. The iLID^{2D}, iLID^{3W} and control mice were injected with MC-38-GFP cells as described in the legend to Figure 1. Shown are representative IHC images seen in liver sections derived from the indicated mice 3 days post injection of 5x10^5 GFP-tagged MC-38 cells (in green). Desmin (in red) and α-SMA (in grey) with DAPI (blue) staining were used to identify and quantify recruited and activated HSC (arrows) as seen in Figure 1.

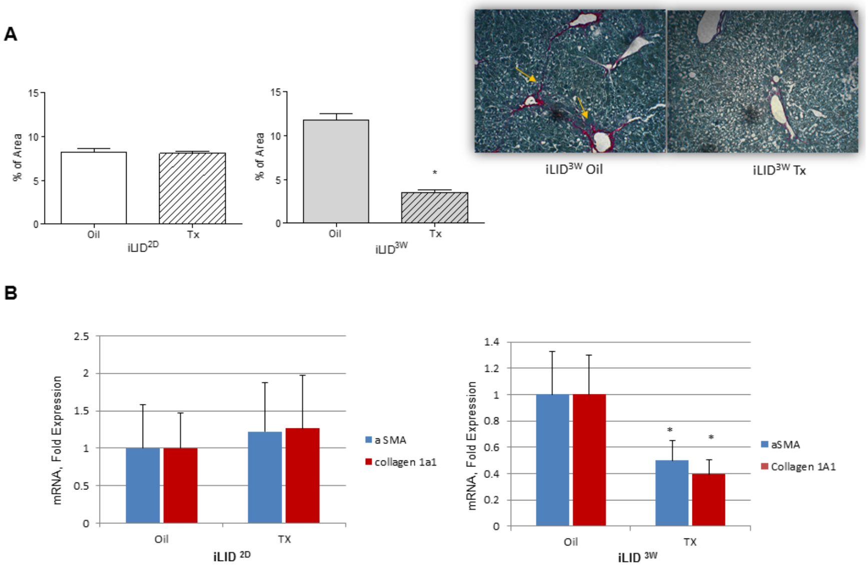

Supplementary Figure S2: A sustained IGF-I deficiency also inhibits carbon tetrachloride (CCL_4)-induced fibrosis. iLID mice were injected with Tx or Oil 2 days (iLID^{2D}) or 3 weeks (iLID^{3W}) prior to initiation of treatment with CCl_4 (25% in sunflower oil) twice weekly for 6 week. FFPE sections **A.** of the livers obtained from CCl_4 treated mice were stained with Sirius Red and the red-stained areas (as indicate by arrows) were quantified in a total of 20-50 fields derived from 3 - 4 animals per condition (x10 objective). Results of the quantification performed by Image J (A-left) are expressed as the % of total surface area/field that stained red (collagen) and representative images (A-right**)** of Sirius Red –stained sections are shown for iLID^{3W} Oil and Tx. A liver fragment was snap frozen and stored at -80°C to perform qPCR **B.** as described in Materials and Methods. Expression levels of two pro-fibrogenic genes (α-SMA and Collagen 1A1) was assessed and the results are expressed as fold change relative to GAPDH. Bar graphs are means (±SEM) of triplicate samples in two independent experiments. *$p<0.05$

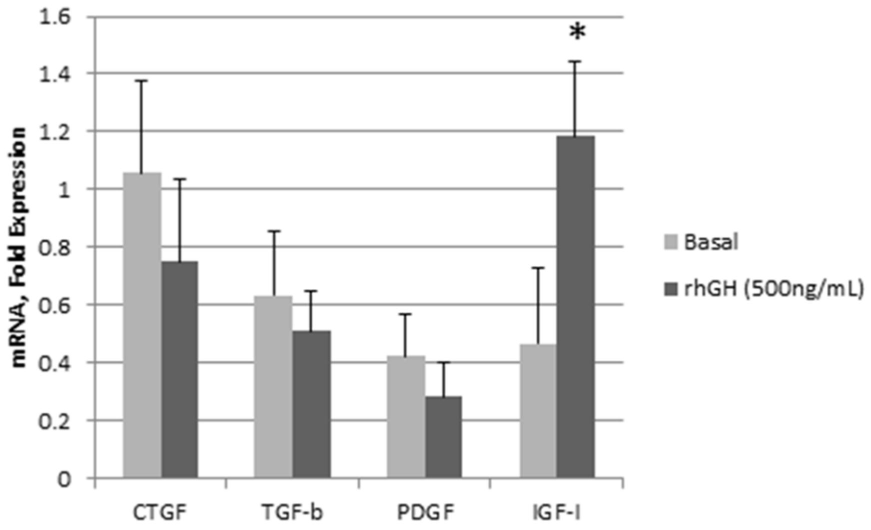

Supplementary Figure S3: GH treatment does not alter the expression of pro-fibrotic genes in colon carcinoma MC-38 cells. Cells were seeded at a density of $1x10^6$ cells per well in 6 well plates and culture in 10% FBS DMEM overnight to allow attachment. The cells were then serum starved overnight and stimulated with 500 ng/ml rhGH for 4 hr. RNA was extracted and qPCR performed using the primers listed in Supplementary Table S2 . Results in the bar graphs are based on triplicate samples per condition in two independent experiments and are expressed as means (±SEM). *p<0.05

Supplementary Table S1: List of Antibodies used in this study. Listed are all antibodies used in this study, their origin, suppliers and the dilutions used

Immunogen	Supplier	Dilution used	Species
Desmin	Dako (ON, Canada)	1:200	Mouse anti-human MAb (cross-reacts with mouse)
Desmin	Thermo Scientific (Waltham, MA)	1:100	Rabbit anti-mouse polyclonal
Glial fibrillary acidic protein (GFAP)	Dako	1:200	Rabbit anti-mouse polyclonal
α-SMA	Dako (clone 1A4)	1:200	Mouse anti-mouse monoclonal
α-SMA	Abcam (Cambridge, UK)	1:200	Rabbit anti-mouse polyclonal
β-actin	Sigma-Aldrich	1:25000	Mouse anti-mouse monoclonal
Alexa Fluor 647	Molecular Probes (Eugene, OR).	1:200	Goat anti-rabbit
Alex Fluor 568	Molecular Probes	1:200	Goat anti-mouse
STAT5a-2H2	Invitrogen (Waltham, MA)	1:1000	Mouse anti-mouse monoclonal
Phospho-STAT5 pTyr694	Invitrogen	1:1000	Rabbit anti-mouse polyclonal
Phospho IGF-IR (Y1161)	Abcam	1:100	Rabbit anti-mouse polyclonal
IGF-IR	Abcam	1:500	Rabbit anti-mouse polyclonal
p44/42 MAPK, Erk1/2 (Thr202, Tyr204)	Cell Signaling Technology (Danvers, MA)	1:1000	Rabbit anti-mouse polyclonal
p44 MAP Kinase (Erk1)	Cell Signaling Technology	1:1000	Rabbit anti-mouse polyclonal
Phospho AKT (Ser473)	Cell Signaling Technology	1:200	Rabbit anti-mouse polyclonal
AKT	Cell Signaling Technology	1:1000	Rabbit anti-mouse polyclonal
Peroxidase conjugated IgG	Jackson ImmunoResearch Laboratories (West Grove, PA)	1:10000	Goat anti-rabbit
Peroxidase conjugated IgG	Jackson ImmunoResearch Laboratories	1:10000	Goat anti-mouse

Supplementary Table S2: List of qPCR primers. Listed are the sequences 3'-5'of the primers used in this study for qPCR quantification

	Forward	Reverse
α-SMA	TCCTCCCTGGAGAAGAGCTAC	TATGGTGGTTTCGTGGATGC
Collagen 1α1	GCGAAGGCAACAGTCGATTC	CCCAAGTTCCGGTGTGACTC
CTGF	TGCGAAGCTGACCTGGAGGAAA	CCGCAGAACTTAGCCCTGTATG
PDGF	CTGGCTCGAAGTCAGATCCACA	GACTTGTCTCCAAGGCATCCTC
TGF-β	TGATACGCCTGAGTGGCTGTCT	CACAAGAGCAGTGAGCGCTGAA
IGF-I	GTGGATGCTCTTCAGTTCGTGTG	TCCAGTCTCCTCAGATCACAGC
GAPDH	TGTGTCCGTCGTGGATCTGA	TTGCTGTTGAAGTCGCAGGAG
